# Estimation of the Age of the Kashubian-Specific Pathogenic *NPHS2* Variant Responsible for Hereditary Steroid-Resistant Nephrotic Syndrome Points to Its Recent Local Origin

**DOI:** 10.1155/2024/8205102

**Published:** 2024-03-21

**Authors:** M. Jankowski, P. Daca-Roszak, I. Bałasz-Chmielewska, A. Ustaszewski, A. Żurowska, B. S. Lipska-Ziętkiewicz, E. Ziętkiewicz

**Affiliations:** ^1^Department of Biology and Medical Genetics, Medical University of Gdansk, Gdansk, Poland; ^2^Institute of Human Genetics, Polish Academy of Sciences, Poznan, Poland; ^3^Department of Pediatrics, Nephrology and Hypertension, Medical University of Gdansk, Gdansk, Poland; ^4^Centre for Rare Diseases, Medical University of Gdansk, Gdansk, Poland; ^5^Clinical Genetics Unit, Department of Biology and Medical Genetics, Medical University of Gdansk, Gdansk, Poland

## Abstract

Steroid-resistant nephrotic syndrome (SRNS) is a highly heterogenic kidney disorder resulting from genetic abnormalities or immune system dysfunction affecting the establishment and maintenance of the glomerular filtration barrier. The most common cause of genetic SRNS is biallelic pathogenic variants in *NPHS2* gene, especially in individuals with an infantile or childhood onset. The type of the *NPHS2* defect implies the course of the disease and the stage of its onset and differs across populations. In a cohort of Polish patients with SRNS, a unique profile of the disease-related *NPHS2* variants was identified in patients from northern Poland inhabited by Kashubs, a minority West-Slavic ethnic group known for a local increase of the frequency of several pathogenic variants. Among Kashubian families, the compound heterozygotes c.686G>A/c.1032delT and a single c.1032delT homozygote were the only underlying cause of SRNS. The restricted, Kashubian-only pattern of c.1032delT occurrence, suggesting the founder effect, prompted us to conduct a detailed analysis of its haplotype background to estimate the age of the c.1032delT origin. Eight Kashubian SRNS families were genotyped using the Infinium Global Screening Array-24. The haplotype background analysis was performed using an in-house pipeline designed to solve the phase of the heterozygous genotype data. The age of the c.1032delT mutation was calculated using the gamma method based on the genetic length of ancestral haplotypes shared between two or more individuals carrying this variant. The results of our study indicated a very recent origin of the c.1032delT mutation (~240 years). Genetic screening performed in the general Polish population control corroborates the assumption that the mutation occurred on the specific Kashubian haplotype background. The identification of ancestry-specific Kashubian pathogenic variant can help to develop effective screening and diagnostic strategies as a part of personalized medicine approach in the region.

## 1. Introduction

Nephrotic syndrome (NS) is a rare kidney disease clinically characterized by severe proteinuria, resulting in complications such as hypoalbuminemia, hyperlipidemia, and edema. Kidney biopsies typically show focal segmental glomerulosclerosis. The first-line treatment is based on steroid administration; however, approximately 10-15% of patients do not respond to immunosuppressive treatment and progress to steroid-resistant nephrotic syndrome (SRNS) [1]. In a subset of patients with SRNS (up to 30%), the genetic etiology of the disease has been identified [2].

SRNS is a highly heterogenic genetic entity with more than 60 genes identified so far. The disease may be caused by pathogenic variants in different podocyte-specific genes, involved in the structure and function of the slit diaphragm (e.g., *NPHS1, NPHS2*), the actin cytoskeleton (e.g., *ACTN4, INF2*), or podocyte differentiation (*WT1*) (for details see [3–5]). Biallelic pathogenic variants in the *NPHS2* gene are the most common causes of the genetic forms of SRNS, especially in those with an infantile or childhood onset [1, 6, 7]. Steroid-resistant nephrotic syndrome due to *NPHS2* variants is not associated with posttransplant recurrence [8].

The *NPHS2* gene is located on chromosome 1q25.2, consists of eight exons, and encodes podocin, a protein almost exclusively expressed in the podocytes of fetal and mature kidney glomeruli. Podocin is a cell-membrane protein with a hairpin-like topology, with both N- and C-terminal domains facing the cytoplasm, located at the insertion of the slit diaphragm in the renal glomerulus. Podocin links nephrin, the slit diaphragm protein, to the cytoskeleton, and thus is crucial in the establishment of the glomerular filtration barrier [9]. Half of over 670 *NPHS2* variants reported to date are classified as pathogenic (*n* = 285) or likely pathogenic (*n* = 53) (after Varsome [10]; accessed May 15th, 2023). Pathogenic *NPHS2* variants, mainly missense, nonsense, and frameshift mutations, are estimated to account for 10–30% of SRNS cases, depending on the ethnicity, size of the analyzed group, accuracy of the clinical diagnosis, and the mean age at diagnosis [11]. They are a common cause of SRNS in Europe, the Americas, Africa, and partly in India [5, 11] but are very rare among SRNS patients in Asia (especially in East Asia), where pathogenic variants in the *COQ8B* gene predominate [12].

The spectrum of *NPHS2* variants involved in SRNS pathogenesis differs across populations. The frequency of c.413G>A (p.Arg138Gln), the prevalent disease-causing *NPHS2* variant in Europeans [11], ranges from 1.3 to 9.3% of SRNS chromosomes in various Western and Central European populations [13–20]. A common variant c.686G>A (p.Arg229Gln), prevalent in European, Indian, and South American populations, is considered a risk factor only if *in trans* with certain pathogenic *NPHS2* variants located in exons 7 and 8 [21, 22]. Compound heterozygosity for the c.686G>A is associated with the later onset of the disease [21, 23]; its frequency ranges from 2.3 to 11% of SRNS chromosomes [13–17, 24]. Other pathogenic *NPHS2* variants have varying, usually low frequencies among SRNS chromosomes. However, a local increase in the occurrence of some of the pathogenic *NPHS2* variants in certain populations is observed, suggesting the presence of a number of founder effects (summarized in Table 1).

In our earlier study performed in a cohort of 141 Polish patients with SRNS [23], twenty patients (14% of the cohort) have fulfilled the criteria of *NPHS2*-associated SRNS. The analysis has revealed a specific profile of the disease-related *NPHS2* variants. Five cases had homozygous mutations (including four with the prevalent European c.413G>A variant), and fifteen were compound heterozygotes. 11 of these harbored the nonneutral polymorphism c.686G>A, transassociated with c.1032delT. Interestingly, the rare c.1032delT variant (not reported in the gnomAD Exomes 2.1.1 database) has been found only in patients from the region of Pomerania in northern Poland. This region is inhabited by Kashubs, a minority West-Slavic ethnic group known for a local increase of the frequency of several pathogenic variants, currently making up to 10-30% of local population [34, 35]. The restricted pattern of c.1032delT occurrence, suggesting the founder effect underlying the local increase of its frequency, prompted us to conduct a detailed analysis of its haplotype background to estimate the age of the c.1032delT origin. For comparative purposes, the analysis of the haplotype background of the c.686G>A variant transassociated with c.1032delT in SRNS patients was performed.

## 2. Material and Methods

### 2.1. Participants

**Figure 1:**
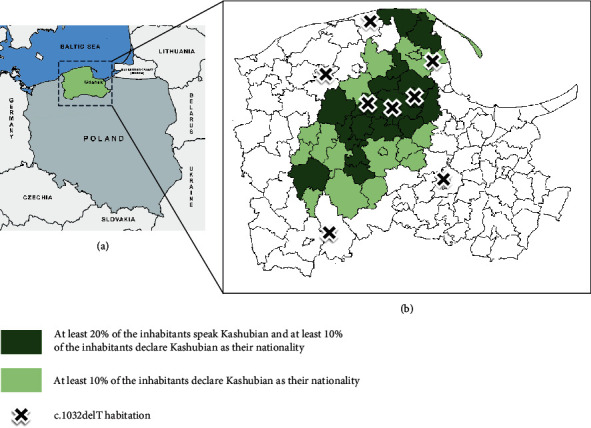
Geographical distribution of c.1032delT Kashubian families in the Pomeranian Region of Poland. (a) Localization of the region at the southern coast of the Baltic Sea in Central-Eastern Europe. (b) Amplified view of the region; the scale of green intensity indicates communities where Kashubs are presently the prevalent ethnic group (data from Central Statistical Office of Poland).

The analysis was performed in eight Kashubian SRNS families (Figure 1 and Table 2), earlier identified to carry the c.1032delT *NPHS2* variant (see [23] for the clinical characteristics of the patients). All Kashubian patients, except for one (patient 4) from the acknowledged consanguineous union, with the c.1032delT present on both alleles, were compound heterozygotes (*in trans*, confirmed by testing available parents) for the c.1032delT and c.686G>A variants. Among the families, two were trios (patient, mother, and father), four consisted of a patient and a single parent, and two were single patients (including patient 4 for whom a single chromosome was counted). Eight chromosomes with c.686G>A in compound heterozygosity with c.1032delT (seven from probands and one from the affected father of patient 2) were used to infer the background c.686G>A haplotype. Four non-Kashubian individuals (with persistent proteinuria) homozygous for the nonneutral c.686G>A *NPHS2* polymorphism were also included in this analysis: two were from Poland (coming from adjacent regions in Western and Central Poland), and two were from non-Polish populations (Turkish and South American of Indian/Hispanic descent). A control group with no pathogenic variants in *NHPS2* consisted of unrelated individuals originating from various parts of Poland: 50 patients (including eight Kashubians) had other hereditary kidney disease (12 caused by *NUP93* biallelic defect, 38 Alport disease); other 50 (including one Kashubian) had no signs of a renal disease.

DNA from SRNS patients and families, and from control nephrological patients (*n* = 50), was obtained from the repository of the Department of Biology and Medical Genetics, Medical University of Gdansk; DNA from a nonnephrological control group (*n* = 50) was obtained from the repository of the Department of Molecular and Clinical Genetics, Institute of Human Genetics PAS.

Patients'/parents' informed consent was obtained. The study was approved by the Ethical Committee of the Medical University of Gdansk, Poland (NKBBN/631/2018).

### 2.2. Genotyping

Genotyping of over 766 thousand SNPs was performed using the Infinium Global Screening Array-24 Kit (Illumina) and Illumina iScan scanner. Normalized signal intensity and genotype were computed using IlluminaBeadArrayFiles Python library and IlluminaBeadArrayFiles.

To extract the relevant information, microarray data on chromosome 1 were filtered to exclude the following: SNPs that were homozygous across the whole study group (including controls); positions with incomplete genotyping data; positions with very rare SNPs not present in SRNS patients and found in less than 10% of the control group chromosomes; positions with very rare SNPs not found in any of the controls and present in a singular SRNS chromosome (presumably representing genotyping errors or recent mutations, but not recombination events). The remaining SNPs surrounding the *NPHS2* gene were subjected to haplotype analysis.

### 2.3. Ancestral Haplotype Analysis

The ancestral haplotype analysis primarily aimed at explaining the background on which the c.1032delT mutation originated; the phase of the c.686G>A variant, transassociated with c.1032delT in SRNS patients or present in homozygous state in four additional individuals, was analyzed for comparative purposes. The analysis was performed using an in-house pipeline designed to solve the phase of the heterozygous genotype data, to extract the single background haplotypes associated with each of the two analyzed pathogenic variants. The main assumptions of the phase solving are presented below.

Solving the phase of the c.1032delT background haplotype was based on the available family data: the heterozygous positions were solved according to the consistency with the parental chromosome carrying the same mutation. When no parental data were available or when all family members were heterozygous for a given SNP position, identity by descent was assumed, and the majority rule was applied (SNP alleles were assumed to be consistent with the majority of the solved haplotypes carrying c.1032delT). The haplotype observed in the homozygous c.1032delT from a consanguineous union (patient 4) was used as an additional indicator for solving c.1032delT background haplotypes in the vicinity of the mutation (up to the point where allele sharing was higher among other individuals harboring c.1032delT). Heterozygous positions not solved by the family data or by the majority rule were considered uninformative and were excluded from further haplotype analysis. Few SNP positions, at which the shared haplotype was interrupted by a single discordant allele followed by another large run of continuous sharing, were assumed to represent genotyping errors or recent mutations (see, e.g., [36]). Solving the c.686G>A background haplotype in heterozygous NPHS2 patients was done by subtracting the c.1032delT haplotype; in c.686G>A homozygotes, it was based on the majority rule.

The presence of two alleles different from that found on the majority of the mutation-carrying chromosomes, if detected at several consecutive SNP positions, marked the end of the shared background haplotype. This rule was also used to infer the maximal range of the analyzed ancestral haplotype in unrelated control samples (we acknowledge that this could have led to the overestimation of haplotype sharing, but it did not change the overall conclusion).

### 2.4. Estimation of the Age of a Founder Mutation

Ancestral segments in sampled individuals were identified by continuous haplotype sharing between two or more unrelated chromosomes with c.1032delT (seen as allele sharing among consecutive markers surrounding the mutation). The segment lengths were calculated from the genetic map positions of the outermost shared markers (to avoid chance sharing, the endmost concordant alleles present in the general European population at the frequency > 0.6 were considered uninformative). The maximum-likelihood estimate of the mutation age was calculated using the gamma method based on the genetic length of ancestral haplotypes shared between two or more individuals carrying the mutation [36]. The gamma method, designed for small samples with dense marker data, implemented in the online software (https://shiny.wehi.edu.au/rafehi.h/mutation-dating/), can be applied to genealogies in which the data are either independent or correlated. The correlated genealogy (where a subset of chromosomes reaches common ancestry earlier than the most recent common ancestor for the entire study group) was assumed for the c.1032delT variant, in accordance with its restricted geographic occurrence and historical distinctiveness of the Kashubian population. The age of the c.686G>A variant (frequent in many European populations) was analyzed in frame of the independent genealogy.

## 3. Results

**Figure 2:**
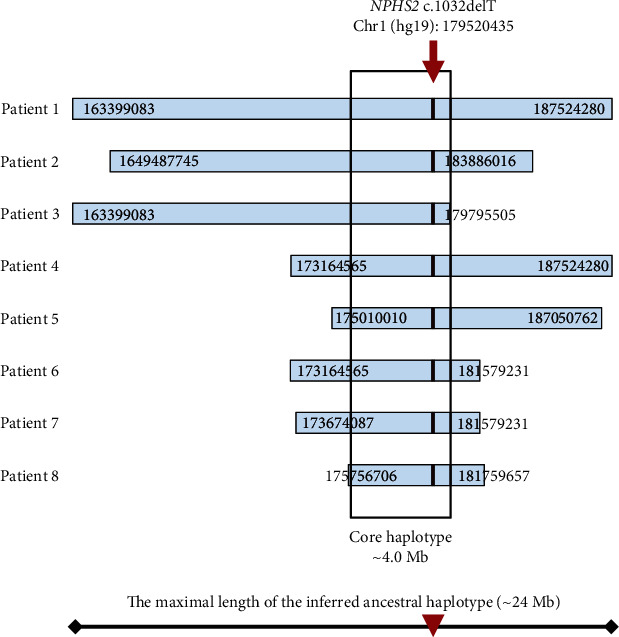
Schematic presentation of the shared haplotypes in the analyzed chromosomes carrying the c.1032delT variant. The maximal length of the inferred ancestral haplotype was ~24 Mb. Frame indicates the core segment of the ancestral haplotype, shared by all the chromosomes carrying c.1032delT mutation.

SNP variability in the studied cohort was analyzed in the area of approx. 30 Mb surrounding the *NPHS2* gene (Figure 2; also see Supplementary Table (available here)). The core haplotype of ~4.0 Mb, shared by all chromosomes carrying the c.1032delT variant, indicated a common origin of the variant in all the examined patients. The length of the haplotype shared by at least two of the chromosomes on any side of the c.1032delT (~24.2 Mb) was assumed to represent the ancestral background on which the mutation had occurred.

The age of the ancestral haplotype carrying c.1032delT mutation was estimated at 12.3 generations assuming both correlated or independent genealogy of the analyzed chromosomes. Assuming the mean time between generations of 20 years, this corresponded to the time of the mutation origin between 240 (CI 140-400) years ago. Of note, the c.1032delT ancestral haplotype of the comparable length was not observed in any among 200 control chromosomes (including those identified as Kashubian); the longest potentially compatible segments observed in four chromosomes (including one of eight Kashubian) were less than 2.5 Mb long (data not shown).

The analysis of the haplotype background of c.686G>A variant revealed a different story. The length of the core haplotype shared by all the chromosomes carrying this variant was very short (~0.51 Mb, see Supplementary material). The maximal length of the identical haplotype shared by at least two of the Kashubian chromosomes, on any side of the c.686G>A, was assumed to represent the common ancestral background on which the mutation entered this population; it was estimated as ~3.3 Mb, which would result in the estimated age of ~3.1 thousand years. Alternatively, the presence of independent mutations on different haplotype backgrounds could be suggested; however, because of the uncertainty of haplotype solving in homozygous individuals from various populations, this was not pursued any further.

## 4. Discussion

Among Kashubian families, the compound heterozygotes c.686G>A; 1032delT and a single c.1032delT homozygote were the only underlying cause of SRNS identified in our studies [23]. The estimated age of c.1032delT (~240 years) indicated a very recent origin of this mutation. Kashubs are a unique ethnic group, descended from West-Slavs tribes, living in the region of Pomerania in northern Poland. Due to various historical and demographical events, Kashubs have developed and retained their separate identity, language, and culture [35, 37, 38]. Kashubs' singularity is also evident in the genetic profile, which differs from that in the neighboring regions of Poland. Studies on the neutral genetic variation (e.g., mtDNA and Y chromosome) have shown that the contemporary genetic profile of Kashubs in eastern Pomerania differs from that in the neighboring regions [39, 40]. The genetic distinctiveness of Kashubs is demonstrated by the increased or decreased frequency of certain variants in the region inhabited by Kashubs compared to other parts of Poland and Europe. It is also seen in the prevalence of some rare genetic diseases (reviewed in [34]): familial hypercholesterolemia, hereditary breast and ovarian cancer syndrome, and long-chain 3-hydroxyacyl-CoA dehydrogenase deficiency; steroid-resistant nephrotic syndrome caused by biallelic defect in the *NPHS2* gene is another example.

The dissemination of c.1032delT only within the Kashubian population may be associated with short-range migrations within the region (from small settlements to larger neighboring towns) at the turn of XVIII and XIX centuries. Interestingly, the present-day geographical location of the individuals carrying c.1032delT mutation and the information on the family origins collected through the individual interviews narrows the origins of the mutation to the region of Kaszëbskô Szwajcariô, a land of numerous glacial lakes located in the middle of the Central European lowland south-west of Gdańsk.

Besides Kashubs, the c.1032del allele in the *NPHS2* gene has been reported in one Polish SRNS patient from the southern region of the country (Cracow) (Lipska-Ziętkiewicz, personal communication), in one Caucasian living in Lubeck, Germany [6], and in two siblings living in the UK, apparently related to one of the Kashubian families analyzed here and described in our previous report [23, 41]. With Germany and the UK being the most common destinations for the recent migrations from Eastern European countries, it is likely that these individuals are actually immigrants of the Kashubian origin.

Genetic screening performed in almost 600 consecutive neonates from Northern Poland [23] has revealed a single carrier of c.1032delT. This variant has not been reported in any of the databases where European population data can be found, indicating that it had occurred in the Kashubian population. Moreover, the lack of the c.1032delT ancestral haplotype (even of its shortest core version) among 100 control Polish chromosomes corroborates the assumption that the mutation occurred on the specific Kashubian haplotype background. The longest segments potentially concordant with the ancestral c.1032delT haplotype, inferred in four control chromosomes, were much shorter (1.6-2.5 Mb) compared to the 4.5 Mb of the core segment shared by all the chromosomes carrying c.1032delT. It should be emphasized that, in the absence of family data in the control group, the inference of the ancestral haplotype indicated the maximal length of the potential concordance with the c.1032delT background; in fact, the ancestral segment in controls could have been much shorter, which would only strengthen our conclusion that c.1032delT occurred very recently on a unique Kashubian background.

Unlike in the case of c.1032delT, a very short segment of the haplotype shared by chromosomes carrying c.686G>A suggested either the very old age of the c.686G>A mutation (in the range of ~3 thousand years) or its recurrent origin. Both scenarios are consistent with the pan-European spread of that variant. In the study of c.686G>A (p.Arg229Gln) [10], the analysis of several markers, three informative SNPs (rs12406197; rs12401708; rs1410592) localized within the *NPHS2* gene and five nearby microsatellites (DS1S3758; D1S3760; D1S215; D1S3759; D1S2883, spanning 1.1 cM and flanking *NPHS2* [13]), revealed the same haplotype on all the examined chromosomes with the c.686G>A allele. This has been reported as indicating the shared origin of the variant [10]. However, we performed the analysis of the three aforementioned SNPs using the LDhap tool implemented in LDlink [42], which indicated that the same minihaplotype (T-T-T on the sense strand) can be expected in 0.23% of control European chromosomes; we did not reexamine the microsatellite markers, but we assumed that the same set of repeats might be present among healthy chromosomes. Sharing of the short haplotype that is relatively frequent also in control chromosomes does not exclude the possibility that the c.686G>A allele is the result of a recurrent mutation. In this context, it should be mentioned that the c.686G>A substitution is located within the CpG dinucleotide. C>T and G>A transitions within CpG dinucleotides, characterized by several times faster mutation rates (related to cytosine methylation), are long recognized as mutation hotspots in a variety of human diseases [43]. This is consistent with the scenario of a recurrent origin of the c.686G>A variant and may explain its high frequency in various populations, with the average of 3.5% observed in most European populations [21] and ~6.5% in Kashubs [23].

An increased homozygosity with respect to founder mutations is usually observed in populations, in which a single pathogenic variant predominates. It is worth noting that the Kashubian-specific c.1032delT variant was not associated with the increased frequency of homozygotes among SRNS patients. The single c.1032delT homozygote was identified in the family with the acknowledged consanguinity, but otherwise, all the patients with c.1032delT were compound heterozygotes with the relatively frequent c.686A>G.

**Figure 3:**
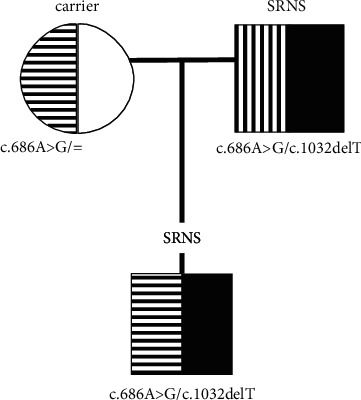
Pseudodominant inheritance of c.1032delT in the family of patient 2, related to the high frequency of c.686G>A in a population.

It has been shown that the pathogenicity of c.686G>A depends on the transassociated mutation in *NPHS2*; it leads to a disease phenotype only when associated with certain 3′ end variants because of an altered heterodimerization and mislocalization of the encoded p.Arg229Gln podocin [22]. The fact that homozygotes of c.686G>A are not clinically affected has an additional impact on SRNS diagnostics. This interallelic interaction results in the incomplete penetrance of c.686G>A [44]. The high frequency of the c.686G>A, notorious for its translocus dependent pathogenicity, in a population increases the risk of a pseudodominant inheritance of rare pathogenic variants, like c.1032delT. This can be exemplified by the segregation observed in one of the analyzed SRNS families (family 2), where unlike in the majority of recessively inherited diseases, both father and son were affected (Figure 3). They both were compound heterozygotes of c.1032delT and c.686G>A, but c.686G>A in the son was inherited from the nonaffected mother. The same can be expected in other populations and other disorders, e.g., Alport disease [45].

## 5. Conclusions

Identification of ancestry-specific pathogenic variants (founder mutations) is important for diagnostic and prevention strategies, e.g., development of effective screening methods [46]. In addition, the estimation of the age and the origin of pathogenic mutations, performed based on the analysis of their background haplotype, sheds light on the historical processes that affected populations.

## Figures and Tables

**Table 1:** Pathogenic *NPHS2* variants characterized by the locally increased frequencies.

cDNA (NM_014625.4)	Protein	Genotypes	Rs ID (ClinVar)	Population with the increased frequency	Variant's worldwide frequency in gnomAD exomes 2.1.1	References
c.353C>T	p.Pro118Leu	Mostly homozygotes	rs869025495 (VCV000222762.5)	Turkey	Not found	[1, 11]
c.412C>T	p.Arg138Ter	Homozygotes and compound heterozygotes	rs74315343 (VCV000005361.14)	Israeli Arab	0.0000159 (African 0.0000616)	[25, 26]
c.413G>A	p.Arg138Gln	Homozygotes and compound heterozygotes	rs74315342 (VCV000005360.52)	Western and Central Europe	0.000597 (European^∗^ 0.00121)	[11]
c.419del	p.Gly140Asp fsTer41	Homozygotes and compound heterozygotes	rs749779208 (VCV000928542.7)	Mediterranean countries such as France, Italy, Turkey, and Cyprus	0.00000796 (European^∗^ 0.0000176)	[27, 28]
c.538G>A	p.Val180Met	Mostly homozygotes	rs74315347 (VCV000005368.17)	North Africa	0.0000119 (Middle Eastern 0.00316)	[11]
c.596dupA	p.Asn199Lys fsTer14	Mostly homozygotes	Not reported	North Africa (Egypt)	Not found	[29]
c.779 T>A	p.Val260Glu	Mostly homozygotes	rs775006954 (VCV000447882.15)	Comoros, Oman, South Africa	0.0000159 (African 0.000246)	[11, 30]
c.851C>T	p.Ala284Val	Mostly assoc. with p.Arg229Gln (c.686G>A)	rs780761368 (VCV000562398.11)	Spain, Portugal, South America	0.000004 (European^∗^ 0.00000886)	[21, 31–33]
c.868G>A	p.Val290Met	Mostly compound heterozygotes	rs200482683 (VCV000190610.17)	Central Europe (Poland, Hungary, Germany, and Turkey)	0.000112 (European^∗^ 0.000221)	[18]
c.1032delT	p.Phe344Leu fsTer3	Mostly assoc. with p.Arg229Gln (c.686G>A)	n.a. (VCV001068472.4)	Kashubs (Poland)	Not found	[23]

^∗^Non-Finnish.

**Table 2:** Summary of analyzed SRNS families.

Patient	Genotype	Gender	Paternal alleles	Maternal alleles
1	c.1032delT/c.686G>A	f	**c.1032delT**	c.686G>A
2	c.1032delT/c.686G>A	m	**c.1032delT/c.686G>A**	c.686G>A
3	c.1032delT/c.686G>A	f	**c.1032delT**	c.686G>A
4	c.1032delT/c.1032delT^∗^	f	c.1032delT	c.1032delT
5	c.1032delT/c.686G>A	f	**c.686G>A**	**c.1032delT**
6	c.1032delT/c.686G>A	f	na	na
7	c.1032delT/c.686G>A	m	c.1032delT	**c.686G>A**
8	c.1032delT/c.686G>A	f	c.1032delT	**c.686G>A**

Parental alleles with the available haplotype data are indicated in bold. The c.686G>A allele not transferred to the affected child is underlined. An asterisk indicates the genotype from the consanguineous union, counted as a single allele. f: female; m: male.

## Data Availability

The allelic composition of the inferred background haplotypes of both analyzed pathogenic variants is provided in the supplementary table. The individual patients' data are not publicly available due to their information that could compromise the privacy of research participants; further data that support the findings of this study are available on request from the corresponding author, E.Z.

## Supplementary Materials

Supplementary Materials Supplementary table reveals the allelic composition of the maximal length of the inferred background haplotypes of both analyzed pathogenic *NPHS2* variants: c.1032delT and c.686G>A.

## References

[1] Trautmann A., Lipska-Ziętkiewicz B. S., Schaefer F. (2018). Exploring the clinical and genetic spectrum of steroid resistant nephrotic syndrome: the PodoNet registry. *Frontiers in Pediatrics*.

[2] Trautmann A., Vivarelli M., Samuel S. (2020). IPNA clinical practice recommendations for the diagnosis and management of children with steroid-resistant nephrotic syndrome. *Pediatric Nephrology*.

[3] Lipska-Zietkiewicz B. S., Adam M. P., Feldman J., Mirzaa G. M. (2021). *Genetic steroid-resistant nephrotic syndrome overview*.

[4] Kopp J. B., Anders H. J., Susztak K. (2020). Podocytopathies. *Nature Reviews: Disease Primers*.

[5] Gbadegesin R., Saleem M., Lipska-Ziętkiewicz B. S., Boyer O., Emma F., Goldstein S. L., Bagga A., Bates C. M., Shroff R. (2022). Genetic basis of nephrotic syndrome. *Pediatric Nephrology*.

[6] Hinkes B. G., Mucha B., Vlangos C. N. (2007). Nephrotic syndrome in the first year of life: two thirds of cases are caused by mutations in 4 genes (NPHS1, NPHS2, WT1, and LAMB2). *Pediatrics*.

[7] Sadowski C. E., Lovric S., Ashraf S. (2015). A single-gene cause in 29.5% of cases of steroid-resistant nephrotic syndrome. *Journal of the American Society of Nephrology*.

[8] Kachmar J., Boyer O., Lipska-Ziętkiewicz B. (2024). Steroid-resistant nephrotic syndrome due to *NPHS2* variants is not associated with posttransplant recurrence. *Kidney International Reports*.

[9] Mulukala S. K. N., Irukuvajjula S. S., Kumar K. (2020). Structural features and oligomeric nature of human podocin domain. *Biochemistry and Biophysics Reports*.

[10] Kopanos C., Tsiolkas V., Kouris A. (2019). VarSome: the human genomic variant search engine. *Bioinformatics*.

[11] Bouchireb K., Boyer O., Gribouval O. (2014). *NPHS2* mutations in steroid-resistant nephrotic syndrome: a mutation update and the associated phenotypic spectrum. *Human Mutation*.

[12] Lu L., Yap Y., Nguyen D. Q. (2022). Multicenter study on the genetics of glomerular diseases among southeast and south Asians: Deciphering Diversities-Renal Asian Genetics Network (DRAGoN). *Clinical Genetics*.

[13] Weber S., Gribouval O., Esquivel E. L. (2004). *NPHS2* mutation analysis shows genetic heterogeneity of steroid-resistant nephrotic syndrome and low post-transplant recurrence. *Kidney International*.

[14] Ruf R. G., Lichtenberger A., Karle S. M. (2004). Patients with mutations in *NPHS2* (podocin) do not respond to standard steroid treatment of nephrotic syndrome. *Journal of the American Society of Nephrology*.

[15] Berdeli A., Mir S., Yavascan O. (2007). *NPHS2* (podicin) mutations in Turkish children with idiopathic nephrotic syndrome. *Pediatric Nephrology*.

[16] Caridi G., Gigante M., Ravani P. (2009). Clinical features and long-term outcome of nephrotic syndrome associated with heterozygous *NPHS1* and *NPHS2* mutations. *Clinical Journal of the American Society of Nephrology*.

[17] Megremis S., Mitsioni A., Mitsioni A. G. (2009). Nucleotide variations in the *NPHS2* gene in Greek children with steroid-resistant nephrotic syndrome. *Genetic Testing and Molecular Biomarkers*.

[18] Kerti A., Csohány R., Szabó A. (2013). *NPHS2* p. V290M mutation in late-onset steroid-resistant nephrotic syndrome. *Pediatric Nephrology*.

[19] Bierzynska A., McCarthy H. J., Soderquest K. (2017). Genomic and clinical profiling of a national nephrotic syndrome cohort advocates a precision medicine approach to disease management. *Kidney International*.

[20] Bezdíčka M., Štolbová Š., Seeman T. (2018). Genetic diagnosis of steroid-resistant nephrotic syndrome in a longitudinal collection of Czech and Slovak patients: a high proportion of causative variants in NUP93. *Pediatric Nephrology*.

[21] Machuca E., Hummel A., Nevo F. (2009). Clinical and epidemiological assessment of steroid-resistant nephrotic syndrome associated with the *NPHS2* R229Q variant. *Kidney International*.

[22] Tory K., Menyhárd D. K., Woerner S. (2014). Mutation-dependent recessive inheritance of *NPHS2*-associated steroid-resistant nephrotic syndrome. *Nature Genetics*.

[23] Lipska B. S., Balasz-Chmielewska I., Morzuch L. (2013). Mutational analysis in podocin-associated hereditary nephrotic syndrome in Polish patients: founder effect in the Kashubian population. *Journal of Applied Genetics*.

[24] Guaragna M. S., Lutaif A. C., Piveta C. S. (2015). *NPHS2* mutations account for only 15% of nephrotic syndrome cases. *BMC Medical Genetics*.

[25] Frishberg Y., Feinstein S., Rinat C. (2006). The heart of children with steroid-resistant nephrotic syndrome: is it all podocin?. *Journal of the American Society of Nephrology*.

[26] Frishberg Y., Rinat C., Megged O., Shapira E., Feinstein S., Raas-Rothschild A. (2002). Mutations in *NPHS2* encoding podocin are a prevalent cause of steroid-resistant nephrotic syndrome among Israeli-Arab children. *Journal of the American Society of Nephrology*.

[27] Özçakar Z. B., Cengiz F. B., Çakar N. (2006). Analysis of *NPHS2* mutations in Turkish steroid-resistant nephrotic syndrome patients. *Pediatric Nephrology*.

[28] Voskarides K., Makariou C., Papagregoriou G. (2008). *NPHS2* screening with SURVEYOR in Hellenic children with steroid-resistant nephrotic syndrome. *Pediatric Nephrology*.

[29] Thomas M. M., Ahmed H. M., El-Dessouky S. H., Ramadan A., Botrous O. E., Abdel-Hamid M. S. (2022). Spectrum of *NPHS1* and *NPHS2* variants in Egyptian children with focal segmental glomerular sclerosis: identification of six novel variants and founder effect. *Molecular Genetics and Genomics*.

[30] Nandlal L., Winkler C. A., Bhimma R. (2022). Causal and putative pathogenic mutations identified in 39% of children with primary steroid-resistant nephrotic syndrome in South Africa. *European Journal of Pediatrics*.

[31] Chernin G., Heeringa S. F., Gbadegesin R. (2008). Low prevalence of *NPHS2* mutations in African American children with steroid-resistant nephrotic syndrome. *Pediatric Nephrology*.

[32] Tonna S. J., Needham A., Polu K. (2008). *NPHS2* variation in focal and segmental glomerulosclerosis. *BMC Nephrology*.

[33] Santín S., Bullich G., Tazón-Vega B. (2011). Clinical utility of genetic testing in children and adults with steroid-resistant nephrotic syndrome. *Clinical journal of the American Society of Nephrology: CJASN*.

[34] Jankowski M., Daca-Roszak P., Obracht-Prondzyński C., Płoski R., Lipska-Ziętkiewicz B. S., Ziętkiewicz E. (2022). Genetic diversity in Kashubs: the regional increase in the frequency of several disease-causing variants. *Journal of Applied Genetics*.

[35] Obracht-Prondzyński C., Wicherkiewicz T. (2012). *The Kashubs: Past and Present. Edited collection VIII, 1–299*.

[36] Gandolfo L. C., Bahlo M., Speed T. P. (2014). Dating rare mutations from small samples with dense marker data. *Genetics*.

[37] Labuda G. (2006). *Historia Kaszubów w dziejach Pomorza, Part 1*.

[38] Mordawski L. (2017). *Atlas dziejów Pomorza i jego mieszkańców – Kaszubów*.

[39] Mielnik-Sikorska M., Daca P., Malyarchuk B. (2013). The history of Slavs inferred from complete mitochondrial genome sequences. *PLoS One*.

[40] Rębała K., Martínez-Cruz B., Tönjes A. (2013). Contemporary paternal genetic landscape of Polish and German populations: from early medieval Slavic expansion to post-World War II resettlements. *European Journal of Human Genetics*.

[41] McCarthy H. J., Bierzynska A., Wherlock M. (2013). Simultaneous sequencing of 24 genes associated with steroid-resistant nephrotic syndrome. *Clinical Journal of the American Society of Nephrology: CJASN*.

[42] Machiela M. J., Chanock S. J. (2015). LDlink: a web-based application for exploring population-specific haplotype structure and linking correlated alleles of possible functional variants. *Bioinformatics*.

[43] Cooper D. N., Youssoufian H. (1988). The CpG dinucleotide and human genetic disease. *Human Genetics*.

[44] Mikó Á., Kaposi A., Schnabel K., Seidl D., Tory K. (2021). Identification of incompletely penetrant variants and interallelic interactions in autosomal recessive disorders by a population-genetic approach. *Human Mutation*.

[45] Mohamed M., Tellez J., Bergmann C., Gale D. P., Sayer J. A., Olinger E. (2022). Pseudodominant Alport syndrome caused by pathogenic homozygous and compound heterozygous COL4A3 splicing variants. *Annals of Human Genetics*.

[46] Shaw T., Chan S. H., Teo J. X. (2021). Investigation into the origins of an ancient BRCA1 founder mutation identified among Chinese families in Singapore. *International Journal of Cancer*.

