# Supplementary material for: Estimation of the Age of the Kashubian-Specific Pathogenic NPHS2 Variant Responsible for Hereditary Steroid-Resistant Nephrotic Syndrome Points to Its Recent Local Origin
Source: Hum Mutat. 2024 Mar 21;2024:8205102. doi: 10.1155/2024/8205102 (PMC11918915; doi:10.1155/2024/8205102)
Supplement: Supplementary Materials — Supplementary table reveals the allelic composition of the maximal length of the inferred background haplotypes of both analyzed pathogenic NPHS2 variants: c.1032delT and c.686G>A. [file 8205102.f1.pdf]

Supplementary Table. Details of the background haplotypes

SNP positions of the LONGEST background c.1032delT (left side) or c.686G>A (right side) haplotypes are shown. For brevity, SNP positions at which alleles in the background c.1032delT haplotype were found in the general population at the frequency >0.6 are not listed. Haplotype segment encompassing the NPHS2 gene (in the reverse orientation) is boxed; red font indicates positions of the studied variants.

SNP name	Pos_hg19	c.1032delT	c.686G>A
rs12735706	163399083	C	
GSA-rs10917756	163422320	G	
rs10753622	163582039	C	
rs10799944	163697998	A	
rs3856207	163747095	A	
rs12139078	163886496	G	
rs7533006	164078275	G	
rs4657329	164082055	C	
rs12037444	164144117	A	
GSA-rs2881506	164212456	G	
rs3898199	164533643	T	
rs2996669	164595489	T	
rs1686184	164663314	C	
rs2171692	164666940	G	
rs1844294	164678924	C	
rs10753644	164691787	G	
rs7543038	164723835	T	
rs12737465	164743778	A	
GSA-rs2185217	164786245	G	
rs10800064	164948745	G	
rs12404676	165032246	T	
rs2881832	165047786	T	
rs2348108	165071376	C	
rs923937	165081033	C	
rs1494405	165107381	A	
rs4657422	165210027	T	
rs71583439	165288280	A	
rs11809911	165288357	C	
rs12057296	165342963	T	
rs12723379	165364340	A	
rs283695	165376499	A	
rs2134095	165377552	G	
rs3835280	165378731	C	
seq_rs10489744	165380623	G	
rs115079560	165383021	C	
rs285480	165403903	G	
rs7519141	165425536	A	
rs56683119	165482885	A	
rs10800120	165605310	A	
rs12723198	165702346	T	
rs61800446	165709420	A	
rs2153264	165787477	G	
rs6677965	165811729	T	
rs4535982	165815939	C	
rs10753694	165818621	A	
rs10494448	165831128	A	
rs4657482	165831649	A	

rs3762365	165860813	C
rs472888	165896757	T
rs2349106	165903962	A
rs6658459	165917032	T
rs9426847	166001463	T
rs1319898	166007024	C
rs10918467	166415259	T
rs12566754	166444011	C
rs7552777	166456704	T
rs7544608	166458160	C
rs6695015	166489143	G
rs530690	166579080	C
rs3845541	166618118	T
GSA-rs530303	166623180	G
rs562445	166730812	T
GSA-rs2064067	166782438	T
rs4656514	166792496	A
rs6675033	166826655	T
rs2075982	166929527	G
rs6698931	167015184	T
GSA-rs1571284	167018916	C
rs6666045	167024631	T
rs12404508	167036714	A
rs2281962	167059760	A
rs988778	167103945	G
GSA-rs11808527	167111293	T
rs869714	167126250	T
rs12409740	167137872	A
rs6663642	167161112	C
rs864537	167411384	G
rs2995093	167445001	G
rs858553	167455145	A
rs17534481	167470817	T
rs2794984	167494622	G
rs704859	167500815	T
rs4657670	167534270	C
GSA-rs11584355	167549747	T
GSA-rs767707	167560542	A
rs1229355	167641292	G
GSA-rs1933075	167676364	A
rs10918751	167702255	A
rs4657718	167772498	A
rs3213588	167778843	G
rs1476075	167794406	A
rs3767457	167822213	C
rs7512378	167825134	G
rs2071921	167825485	C
rs203849	167849414	A
rs11558511	168013850	C
GSA-rs10753767	168167553	A
GSA-rs3819818	168257331	G
rs10800350	168262624	A
rs6427122	168274691	C
rs1883148	168278264	G
rs6685230	168368154	C
rs2300568	168375973	T
rs4656587	168382000	G

GSA-rs1810467	168413221	A
rs858099	168426153	A
rs2206117	168435184	C
rs10918904	168453205	G
rs1323530	168489847	C
rs524705	168665974	C
rs610403	168707974	G
GSA-rs655128	168711459	T
rs2786765	168741270	C
rs6427151	168748125	C
rs10800383	168764740	T
GSA-rs10918996	168795713	T
rs6656076	168815719	A
rs2143091	168816245	G
GSA-rs169266	168824111	T
rs1885555	168851940	G
rs10737544	168891735	G
rs6427160	168892181	T
rs6675509	169043061	A
rs1200106	169056946	A
rs1200114	169060489	G
rs926516	169073050	C
rs1358714	169079419	A
GSA-rs6030	169498975	C
rs6427200	169523706	A
GSA-rs7540556	169531442	C
GSA-rs10489185	169548892	A
rs6703865	169550963	A
GSA-rs1018828	169557734	G
rs6127	169566313	T
rs2235302	169580290	T
GSA-rs3917688	169591080	T
rs1800805	169601281	T
rs1569476	169608917	T
rs10157266	169756389	A
GSA-rs16862682	169757024	T
GSA-rs9803860	169794359	G
GSA-rs10919299	170067296	A
rs12042109	170085453	C
GSA-rs532141	170139508	C
rs11585754	170141722	A
rs7518785	170168182	T
rs34760177	170191520	G
rs4509636	170192022	C
rs2225588	170202563	C
rs983118	170206902	A
rs6427247	170380480	G
rs1591148	170386703	T
GSA-rs12044570	170443579	C
GSA-rs16863425	170519884	C
rs12567093	170554331	C
GSA-rs1928715	170567437	C
rs7540157	170582464	C
rs619456	170585832	C
rs577676	170587340	T
GSA-rs6665169	170605146	T
rs12129225	170615833	C

GSA-rs623752	170618291	**G**
rs502612	170641803	**C**
rs593479	170642899	**C**
rs520131	170654118	**T**
rs17550940	170657335	**C**
rs513287	170664237	**A**
rs10800546	170807586	**A**
rs10919497	170824375	**C**
GSA-rs7517226	170832682	**G**
GSA-rs979070	170897273	**A**
rs4656815	170901445	**G**
rs17562549	170913695	**G**
rs4656233	170915133	**C**
rs28850650	170943197	**C**
rs6608461	171056691	**G**
rs2064076	171062498	**G**
rs12076145	171069959	**A**
rs2266782	171076966	**G**
rs2075992	171080485	**C**
rs1795240	171091875	**A**
rs1736565	171112490	**C**
rs1795244	171119021	**T**
rs11806072	171156997	**G**
GSA-rs10798318	171302986	**A**
rs2235510	171314142	**C**
GSA-rs1963273	171322446	**T**
rs6670432	171324017	**A**
GSA-rs2421809	171371998	**A**
rs2207189	171388916	**C**
rs7519763	171389475	**T**
rs7540116	171393787	**C**
GSA-rs10913039	171417202	**T**
rs235854	171592869	**A**
GSA-rs12029997	171597351	**T**
rs604864	171608466	**C**
rs235913	171618656	**T**
rs9943293	171654968	**T**
rs6685742	171674774	**A**
GSA-rs12032340	171735198	**C**
rs2232818	171755170	**A**
GSA-rs12753344	171799381	**A**
rs10798691	171807956	**A**
rs2224396	171955221	**A**
rs10910863	171960993	**T**
rs12091015	172019718	**T**
rs4075021	172063516	**G**
rs583578	172156886	**G**
rs633995	172186729	**G**
rs12045298	172302258	**C**
GSA-rs9425291	172312769	**G**
rs1011731	172346548	**A**
rs714515	172352990	**A**
GSA-rs9286854	172366806	**G**
GSA-rs10912080	172603411	**C**
rs2859228	172613241	**A**
rs2859229	172615017	**C**
rs1492899	172615395	**C**

rs2859242	172624864	C
rs929087	172632057	G
rs10458360	172633975	C
rs859655	172650450	A
rs859673	172680465	C
rs4916193	172684613	G
rs859637	172711000	T
rs2022168	172724394	G
GSA-rs12145987	172727553	G
rs859627	172728946	G
rs11810332	172789765	A
rs57701379	172810439	T
rs4916288	172884872	G
rs6425189	172930064	C
rs2179424	172935443	A
rs10912561	173164565	T
rs4916319	173266578	A
rs7546568	173297704	A
rs1342038	173301516	A
rs1539259	173312501	G
rs4916352	173369724	C
GSA-rs4916219	173373183	T
rs10798277	173373452	C
GSA-rs72709386	173674087	T
GSA-rs72709386	173674087	T
rs997985	175010010	G
rs6694071	175057782	G
rs12083818	175083952	C
rs6690229	175086461	G
GSA-rs6656284	175087677	A
GSA-rs6696455	175087729	C
rs7521981	175091151	A
rs10798333	175092707	T
rs6701037	175120079	C
rs3753555	175162805	T
rs6659761	175183939	T
GSA-rs61805318	175275465	C
rs11802001	175290855	A
rs3766680	175296448	T
rs9283389	175309281	T
GSA-rs61806376	175312291	G
GSA-rs2236883	175338419	C
GSA-rs1351963	175343297	C
rs10912985	175436833	A
rs859413	175444933	A
rs12738827	175454533	G
GSA-rs2861311	175469011	G
GSA-rs859357	175493901	G
rs859363	175495225	A
GSA-rs78931309	175505444	A
rs17305076	175534602	T
rs4652099	175629207	A
rs12025758	175638336	A
rs9787193	175692304	T
GSA-rs6425361	175756706	T
rs12561903	175757061	T
GSA-rs115043014	175757311	G

GSA-rs78950439	175813072	A	
rs532799	175852209	A	
rs10753110	175869554	C	
GSA-rs352299	176248930	C	
rs2455730	176295837	G	
rs7552857	176429364	G	
rs11583447	176521349	T	
rs12134567	176601365	G	
rs12118102	176634724	G	
rs12117539	176703822	G	
rs1325598	176792249	G	
GSA-rs74826447	176940744	T	
GSA-rs172917	176992553	A	
rs227530	177058201	T	
rs7519595	177074669	T	
rs6667502	177111162	T	
rs12128612	177158631	C	
rs10753144	177165301	G	
rs10913300	177193408	A	
rs932311	177193697	T	
rs16851031	177258204	T	
rs12569209	177279412	A	
rs10798510	177303628	T	
rs9425805	177321917	T	
rs9662701	177384036	G	
rs2038486	177458431	T	
rs1098777	177508044	A	
rs10737325	177512260	T	
rs1252066	177626285	T	
rs17625634	177629723	T	
rs12035381	177648733	G	
rs10753167	177651729	T	
rs4509537	177671478	T	
rs7542551	177680031	A	
rs3131331	177739013	T	
rs10913419	177755464	G	
rs7411090	177762940	C	
rs4072161	177769687	T	
rs10798572	177791110	T	
rs750725	177842998	C	
rs6659428	177863472	C	
rs10798580	177864554	G	A
rs623479	177898799	C	C
rs2182914	177903578	C	T
rs3828137	177920754	A	A
rs10913472	177929554	C	C
GSA-rs41267164	177939593	A	G
rs7340104	177971866	C	T
rs12074053	177974569	T	C
rs7530170	178001395	G	A
GSA-rs75130631	178018302	G	A
rs7540551	178018617	A	G
rs76981544	178019456	C	T
rs73049311	178029856	A	G
rs16852409	178041772	G	A
rs11583320	178042145	C	T
GSA-rs113867106	178191001	C	T

GSA-rs1412784	178206664	G	A
GSA-rs12239670	178377761	T	G
rs4304537	178476554	T	C
GSA-rs41267184	178483355	T	C
rs12036376	178582741	A	G
rs6659392	178598873	T	C
rs10429850	178612000	G	A
rs1122579	178615214	C	T
rs2811314	178619183	A	G
GSA-rs111491640	178659370	A	C
rs1018669	178687459	A	C
rs12217138	178911282	T	C
rs10737335	178943738	T	T
rs1012066	178964604	A	G
rs7534481	178971774	T	C
GSA-rs10913670	178972922	T	C
rs10913672	178981416	G	A
GSA-rs12725164	178984086	C	T
rs6425513	179006462	G	A
rs3829794	179072728	T	T
GSA-rs9728480	179089491	A	C
GSA-rs10913736	179335366	T	C
GSA-rs12725126	179427632	A	G
***NHPS2* 3'**	179519674	T	T
**c.1032delT**	179520435	delA	A
**c.686G>A (rs61747728)**	179526214	C	C>T
***NHPS2* 5'**	179545068	G	G
rs3930537	179553724	A	G
rs10753200	179564144	A	G
rs6704505	179600001	G	A
rs35032839	179704436	G	A
rs2245195	179716507	C	T
rs34831485	179731680	A	C
rs6680679	179753147	G	A
GSA-rs115142749	179762634	C	T
rs2777920	179776841	G	A
rs1148821	179795505	T	C
rs357050	180234729	C	T
rs7536561	180243524	G	G
rs78368507	180338404	T	C
rs10798778	180536954	G	G
GSA-rs7519155	180560692	T	T
GSA-rs116798554	180564319	A	G
GSA-rs77382340	180568640	A	G
rs3843276	180570408	A	A
rs16856367	180645588	A	A
GSA-rs76219649	180652052	G	G
rs12408176	180692815	T	T
rs7536392	180877409	A	A
rs12735084	180893135	C	C
rs3795504	180905448	T	T
rs57113693	180952294	C	C
rs1411478	180962282	A	A
rs10494536	180975865	C	C
rs10797667	181028733	T	T
rs6658698	181060791	G	G
rs6702513	181086122	C	C

rs3897436	181100160	T	T
rs7523821	181112055	G	G
rs4652567	181114794	A	A
rs10752838	181116023	T	T
rs10797679	181149074	G	G
rs10797688	181181995	C	
rs6425678	181185861	G	
rs1004505	181197252	C	
rs10797698	181199735	T	
GSA-rs3911582	181210746	C	
GSA-rs4652630	181227982	A	
rs2501545	181230769	T	
GSA-rs2609500	181270232	A	
GSA-rs2609483	181291866	A	
GSA-rs7548242	181296409	A	
rs9803794	181309400	G	
rs3845428	181311555	T	
rs2804699	181322837	C	
rs2609497	181343029	C	
rs605340	181358028	A	
GSA-rs667586	181372624	A	
rs16857253	181378661	T	
rs67837121	181396050	C	
rs662921	181416024	G	
rs6689905	181428264	G	
GSA-rs7521268	181458840	A	
rs681271	181483786	G	
rs6424815	181507754	T	
rs10910955	181519521	G	
rs1953690	181534435	A	
rs17443123	181548341	G	
rs2332357	181554157	C	
rs199960	181579231	A	
rs10910979	181653111	T	
rs72735234	181697195	G	
rs3767000	181712290	G	
GSA-rs3767003	181716658	G	
rs697259	181736189	A	
seq_rs169235	181740924	G	
rs704326	181759657	G	
GSA-rs610100	181778329	C	
GSA-rs3856109	182069992	T	
rs1689804	182121598	G	
GSA-rs10911032	182185360	T	
rs10494545	182200659	T	
rs2251093	182226964	G	
rs4600010	182262763	A	
rs12142225	182298784	G	
GSA-rs10797768	182312476	A	
rs2252483	182333131	G	
rs12136955	182358944	A	
rs1409051	182377181	A	
rs267855	182387507	G	
rs6692352	182397765	T	
rs6667166	182404101	T	
rs6677117	182410409	G	
rs7535533	182496829	G	

rs579006	182549336	**G**
rs627928	182551337	**C**
GSA-rs3738579	182556036	**G**
rs12041623	182561633	**C**
rs10797788	182623509	**G**
rs2482806	182684087	**A**
rs2275171	182784022	**C**
rs9425569	182942202	**G**
seq_rs10752881	182973491	**A**
rs2483675	183118282	**T**
rs10911269	183134719	**A**
rs519544	183157131	**G**
rs672059	183162539	**G**
rs616545	183298150	**G**
GSA-rs10797875	183298502	**T**
GSA-rs664422	183325722	**C**
rs2022013	183353853	**C**
rs2274064	183542387	**C**
rs10911367	183561517	**T**
GSA-rs34840421	183579729	**T**
GSA-rs4507976	183666920	**G**
rs1926830	183713441	**A**
GSA-rs766721	183761310	**G**
rs4652825	183793323	**G**
rs6424913	183798877	**A**
rs10494569	183802970	**T**
rs3010055	183817289	**T**
rs4651156	183837032	**A**
rs10911457	183843104	**T**
GSA-rs11585126	183850901	**A**
seq_rs10797919	183852914	**G**
GSA-rs10797921	183886016	**A**
rs10494572	183983580	**A**
rs756199	184002874	**G**
rs3814333	184007119	**T**
rs1327124	184014199	**G**
rs1926872	184018475	**C**
rs2274432	184020945	**A**
rs1046934	184023529	**C**
rs10911505	184049978	**C**
rs971572	184068508	**A**
rs10911518	184111365	**A**
rs732812	184113245	**C**
rs6424925	184128264	**A**
rs4650656	184148645	**T**
rs7527771	184149990	**T**
rs10911524	184169492	**G**
rs6696916	184192465	**A**
rs10911551	184241399	**C**
rs932993	184283674	**T**
rs9787399	184294796	**T**
rs2779286	184297478	**G**
rs75488863	184498780	**C**
rs749214	184536171	**T**
rs7540093	184603098	**A**
rs6671374	184621930	**A**
rs3895196	184728874	**T**

GSA-rs682331	184761365	A
rs234644	184833344	G
GSA-rs74705099	184840553	A
rs234660	184852414	G
rs234633	184899649	A
rs10911659	184905261	C
rs7512071	184921581	C
rs170885	184941749	G
rs234117	184951676	T
rs6424976	185317357	T
rs2281415	185392845	T
rs10798014	185394834	G
GSA-rs6685999	185451824	G
rs12128031	185453884	A
rs232298	185455790	C
rs10911748	185497338	T
rs1407277	185500084	G
rs4651268	185512838	G
rs1321996	185515461	A
GSA-rs74455225	185619568	T
GSA-rs115539402	185882740	T
GSA-rs113533719	185985526	A
rs4141459	185995616	C
rs12129650	186026474	T
rs10798035	186050417	G
rs4651295	186054055	G
rs7545010	186126099	G
GSA-rs586727	186155692	T
GSA-rs6676055	186190163	T
GSA-rs1795022	186247924	A
rs1293970	186258229	G
GSA-rs1293989	186267100	A
rs2273779	186273994	C
GSA-rs3131557	186441585	G
rs5275	186643058	G
rs4651331	186817041	G
rs12143166	186859489	G
rs10798069	186875459	G
rs12726519	186898065	T
rs6697145	186910591	T
rs10489409	186920114	C
GSA-rs28395831	186946869	G
rs10798078	186967702	G
rs12139055	186968711	G
rs12566908	186969167	C
GSA-rs55778118	186992276	C
GSA-rs12239390	187000047	G
rs1160832	187013915	A
rs72716034	187027181	G
rs1474864	187033589	T
GSA-rs72716052	187050762	G
GSA-rs10911998	187057847	C
rs1924724	187154470	T
GSA-rs4144116	187267411	A
rs2802474	187328545	G
GSA-rs10912059	187354887	A
rs10798107	187362785	T

rs1339082	187524280	**A**
